# Orphan SelD proteins and selenium-dependent molybdenum hydroxylases

**DOI:** 10.1186/1745-6150-3-4

**Published:** 2008-02-20

**Authors:** Daniel H Haft, William T Self

**Affiliations:** 1 Department of Bioinformatics, J. Craig Venter Institute, Rockville, MD, 20850, USA; 2 Department of Molecular Biology and Microbiology, Burnett School of Biomedical Science, College of Medicine, University of Central Florida, Orlando, FL, 32826, USA

## Abstract

Bacterial and Archaeal cells use selenium structurally in selenouridine-modified tRNAs, in proteins translated with selenocysteine, and in the selenium-dependent molybdenum hydroxylases (SDMH). The first two uses both require the selenophosphate synthetase gene, *selD*. Examining over 500 complete prokaryotic genomes finds *selD *in exactly two species lacking both the selenocysteine and selenouridine systems, *Enterococcus faecalis *and *Haloarcula marismortui*. Surrounding these orphan *selD *genes, forming bidirectional best hits between species, and detectable by Partial Phylogenetic Profiling *vs. selD*, are several candidate molybdenum hydroxylase subunits and accessory proteins. We propose that certain accessory proteins, and orphan *selD *itself, are markers through which new selenium-dependent molybdenum hydroxylases can be found.

**Reviewers:**

This article was reviewed by Arcady Mushegian and Kira Makarova.

## Findings

A considerable number of selenoproteins are known, in which the sulfur (S) atom of the cysteine side chain is replaced by the analogous but much scarcer element selenium (Se), typically at an enzyme active site. This substitution improves enzyme characteristics [1], such that one in four prokaryotic genomes carries a selenocysteine (SeCys) incorporation system [2,3]. SeCys is incorporated during translation at specific UGA codons as the twenty-first amino acid in a system that requires a signal from mRNA secondary structure, a special tRNA (*selC*), the *selA *(in bacteria), *selB*, and *selD *genes [4], and some means of selenium uptake. Species that make even one SeCys-containing protein often make several, with twelve found in *Carboxydothermus hydrogenoformans *[5]. This pattern suggests that the system may be hard to acquire or maintain, in evolutionary terms, but relatively easy to reuse. SelD makes the activated form of selenium, selenophosphate, and is required also in a fundamentally different system, tRNA modification with selenouridine [4,6]. We find the marker specific to that system, 2-selenouridine synthase, in 59% of genomes with SeCys systems but just 11% of those without. Such overlapping usage patterns for selenophosphate could mask additional roles for *selD*.

We searched for orphan (unexplained) *selD *genes in the first 500 complete prokaryotic genomes and found two instances, one in the bacterium *Enterococcus faecalis *V583 and one in the archaeon *Haloarcula marismortui *ATCC 43049. Surrounding the orphan *selD *genes in both species were proteins related by bi-directional best hits (BBH) between the two genomes. These BBH pairs included homologs to subunits of a class of molybdenum cofactor-containing enzymes termed the molybdenum hydroxylases (MH), plus additional uncharacterized proteins and possible maturations proteins. This finding immediately suggested a biological role for the orphan *selD *genes, as there are a few known examples of enzymes with a labile selenium atom, which is contributed not by SeCys but through some unknown mechanism. Such enzymes belong to the MH class, and are designated selenium-dependent molybdenum hydroxylases (SDMH) [7-10]. We noted that various other species carried homologs to several of these BBH hit pairs, typically colocalized with each other, and always in species carrying *selD*. This observation suggests that the SelD protein, orphan or otherwise, may activate selenium for SDMH maturation. Three SDMH activities have been characterized from two species: nicotinic acid hydroxylase and xanthine dehydrogenase from *Eubacterium barkeri *[9,10], and xanthine dehydrogenase and purine hydroxylase from *Clostridium purinolyticum *[7,8]. These species also incorporate SeCys; their genome sequences are unavailable, but must carry *selD*.

The MH contain a catalytically essential, labile (cyanolyzable) sulfur atom, and require shared accessory proteins for enzyme maturation [11]. The labile selenium of the SDMH may have a like need. The few known SDMH act on varied substrates. They may be more similar in sequence to their closest selenium-free homologs than to each other, but likely would share certain selenium insertion proteins. Such accessory proteins, if identified, could serve as genome markers for species producing SeCys-independent selenoenzymes. Several BBH gene pairs in the regions of the two orphan *selD *genes lack homology to any mature enzyme subunit and are candidate SDMH accessory proteins.

The *selD *gene is relatively uncommon, occurring in about 30% of completed prokaryotic genomes. Finding only *selD*-positive genomes far down a list of BLAST matches would strongly suggest a role in selenium metabolism. Recently, we introduced the method of partial phylogenetic profiling (PPP) [12], which can read this signal. PPP is a means to identify rapidly those protein families closely related in their patterns of distribution to a phenotype, a defined protein family, or a calculated Genome Property [2,3]. Phylogenetic profiling techniques, though very powerful in principle [13], rely on good protein families; PPP adjusts the size of each candidate protein family dynamically to find the best match to a profile. For every protein in a query genome, it scores a profile only as large as the working family size (hence "partial") vs. the reference profile, and chooses the size that gives the greatest statistical significance. All genes from a query genome are tested, and ordered according to their individually optimized scores. This method can efficiently rediscover many entire biological systems, such as histidine biosynthesis genes, given a profile based on any one marker gene for the system.

We applied PPP [12] to both the *E. faecalis *[14] and *H. marismortui *[15] genomes, vs. the profile for *selD*, using a skew parameter that favors families containing subsets rather than supersets of the species with *selD *[16]. The top hit in *E. faecalis*, other than *selD *itself, was the adjacent gene EF_2566. Though uncharacterized, it shares N-terminal homology (see Pfam model PF01206) [17] with the sulfurtransferase SirA. The *selD*-dependent species distribution (21 of 21 species) and colocalization with *selD *in five species strongly suggest that this protein acts in selenium metabolism, though its function remains unknown. Its family is now represented by TIGRFAMs model TIGR03527. The next hit, EF_2568, homologous to cysteine desulfurases and selenocysteine lyases, probably is a selenocysteine lyase. The second hit in *H. marismortui*, pNG7234, annotated as a phosphate transporter subunit, probably transports selenate as well [18]. These findings show protein families likely involved in selenium metabolism, probably prior to selenophosphate synthesis, and are not pursued further here.

However, the fifth hit in *E. faecalis*, EF_2563, and the top hit in *H. marismortui*, pNG7241, are members of the same protein family, TIGR03309. They are found close to *selD *and form a BBH pair between the two genomes. The archaeal protein is actually a fusion protein, with its N-terminal half homologous to a xanthine dehydrogenase maturation factor, XdhC [19]. All members of TIGR03309, including YqeB from *Escherchia coli*, are from species with *selD*, but then a sharp fall-off in scores occurs before the next set of homologs is encountered, largely from species that lack *selD*. Reexamination vs. an expanded protein database that includes whole genome shotgun data now confirms that TIGR03309 occurs only in *selD*-positive genomes. Even after removing redundant sequences with greater than 45% sequence identity, 26 species remained, leading to vanishingly small odds that TIGR03309 is not closely connected to selenium metabolism. The additional species include a third with an orphan *selD *gene, *Clostridium phytofermentans*, a cellulose-degrading, ethanol and hydrogen-producing bacterium [20]; the *selD *and TIGR03309 genes are one gene apart.

The restriction of the TIGR03309 protein family to *selD*-positive genomes, frequent fusion with domains homologous to the maturation factor XdhC, and recurring location in extended MH loci suggest that TIGR03309 members mark a broadly distributed biological system connected to SDMH. To search for components of that system, we applied the PPP algorithm to the profile of TIGR03309 distribution. In each genome tested, most of the top hits fell within a single extended locus. Ten of the eleven top hits fall within a 23-gene purine catabolism [21] and selenate reduction [22] region in *Escherichia coli*. Similarly, the eleven top hits in *Aeromonas hydrophila *sit together, as do six of the top seven in *H. marismortui*, seven of nine in *Clostridium difficile*, etc. These regions typically include two additional uncharacterized families, TIGR03310 (YgfJ in *E. coli*) and TIGR03172 (YqeC in *E. coli*). We propose that these families are also SDMH accessory proteins, possibly for a smaller subset of SDMH than for TIGR03309. Table 1 shows how well the top 25 hits by PPP *vs*. the TIGR03309 profile mark regions of conserved gene clustering between *E. coli*,*E. faecalis*, *H. marismortui*, and *C. difficile*.

**Table 1:** Conserved clustering of top Partial Phylogenetic Profiling results

*E. coli *gene	Putative protein name	PPP rank	*E. coli *locus	PPP rank	*E. faecalis *locus	PPP rank	*H. maris*. locus	PPP rank	*C. difficile *locus
xdhA	xanthine dehydrogenase, Mo-binding subunit	6	b2866	14	EF_2570			5	CD2087
xdhB	xanthine dehydrogenase, FAD binding subunit	15	b2867						(CD2101)
xdhC	xanthine dehydrogenase, Fe-S binding subunit	17	b2868	14	(EF_2570)				(CD2088)
ygeV	sigma(54)-dependent activator	13	b2869						
ygeW	carbamoyl transferase	7	b2870	5	EF_2577				
ygeX	diaminopropionate ammonia-lyase	10	b2871	7	EF_2579			7	CD2085
ygeY	hydrolase, peptidase M20 family	4	b2872	3	EF_2578				(CD2084)
ygeZ	phenyl-hydantoinase	19	b2873		(EF_2580)	6	pNG7258		
yqeA	carbamate kinase	18	b2874	15	EF_2575				
**yqeB**	**SDMH accessory protein TIGR03309**	**1**	**b2875**	**2**	**EF_2563**	**1**	**pNG7241**	**1**	**CD3478**
yqeC	SDMH accessory protein TIGR03172	2	b2876	1	EF_2564	2	pNG7237	2	CD2071
ygfJ	SDMH accessory protein TIGR03310	5	b2877	21	EF_2569		pNG7236		
ygfK	selenate reductase, Fe-S subunit	8	b2878	4	EF_2581				
ygfL	selenium metabolism protein SsnA TIGR03314	3	b2879	6	EF_2582	8	pNG7259		
ygfM	selenate reductase, FAD-binding subunit		b2880						
ygfN	selenate reductase, Mo-binding subunit	14	b2881		(EF_2570)	3	pNG7246	**3**	**(*CD2099*)**
						5	(pNG7244)	4	CD2073
								5	(CD2079)
ygfO	xanthine/uracil family permease		b2882		(EF_2573)				
	sulfurtransferase-related protein TIGR03527			12	EF_2566		pNG7238		CD3667
selD	selenophosphate synthase		b1764		EF_2567		pNG7239		CD2496
	selenocysteine lyase TIGR01977			11	EF_2568				CD3670

Partial Phylogenetic Profiling results for *Escherichia coli*, *Enterococcus faecalis*, *Haloarcula marismortui*, and *Clostridium difficile vs*. the profile of the putative SDMH accessory protein family TIGR03309. The first seventeen rows show a contiguous gene region in *E. coli*, and the last three a region in *E. faecalis*. The first two columns show *E. coli *gene symbols and putative protein names. The third, fifth, seventh, and ninth columns represent rank by PPP for *E. coli *(out of 4243 proteins), *E. faecalis *(3277 proteins), *H. marismortui *(4240 proteins – all loci found are on plasmid with 362 proteins), and *C. difficile *(3873 proteins); missing values show that the protein did not score in the top twenty-five. Locus tags shown in columns 6, 8, and 10 mark genes with bi-directional best hits vs. the corresponding *E. coli *locus in column 4 and co-clustered in their respective species, so not all of the top 25 hits appear in the table; locus tags in parentheses are unidirectional best hits from the E. coli gene. The row for putative SDMH accessory protein family TIGR03309 is in boldface. The putative *C. difficile *ortholog to the *C. purinolyticum *xanthine dehydrogenase molybdenum-binding subunit is in italicized boldface.

TIGR03309 clearly is connected both to selenophosphate synthase activity and to molybdenum hydroxylases. In theory, the connection might be that TIGR03309 represents an accessory protein to an apparent selenate reductase found nearby in E. coli, in a selenium detoxification system that requires activation by SelD. Searching for homologs to selenate reductase subunits, however, pulls in few genomes with both TIGR03309 and SelD before hitting large numbers of genomes with neither. Therefore it seems more likely that the selenate reductase helps furnish selenide to SelD in select species, while TIGR03309 family members contribute to SDMH maturation. While no complete genome sequence is available for either genome with characterized SDMH, N-terminal sequences are available for *C. purinolyticum *selenium-dependent xanthine dehydrogenase (XDH) subunits, from which we identify putative orthologs in the nearly complete genome of *C. difficile*. The top hit there, after the TIGR03309 and TIGR03172 family putative SDMH accessory proteins, is the apparent ortholog to the characterized XDH molybdenum-binding subunit. This last observation appears to cement the connection of SDMH with both TIGR03309 and *selD*, and suggests a means to discover new selenium-dependent molybdenum hydroxylases. This class of enzyme may be more common than previously appreciated, and may involve purine catabolic systems in gut bacteria such as *E. coli*. This notion is intriguing because a defect in purine metabolism causes gout; human health therefore may be affected by selenium availability to gut bacteria.

## Competing interests

The author(s) declare that they have no competing interests.

## Authors' contributions

DH performed the analyses. DH and WS interpreted the results. DH wrote the paper. DH and WS edited the paper.

## Reviewers' comments

### Reviewer 1: Arcady Mushegian, Stowers Institute for Medical Research

The note reports several observations that are of interest. I recommend the following modifications.

1. As it is currently written, the report is mostly chronological: it starts with the observations were made first, follows with other observations they led to, etc. I suppose that the notes on computational prediction may benefit from following a different plan, which is a bit more formal: first state all predictions that you are making, then show what is the evidence supporting these predictions, and then tell why alternative explanations of the same evidence are less likely than the initial hypothesis. This will make the logic much easier to follow.

2. This work: Lobanov AV, Hatfield DL, Gladyshev VN. Selenoproteinless animals: Selenophosphate synthetase SPS1 functions in a pathway unrelated to selenocysteine biosynthesis. Protein Sci. 2008 Jan;17(1):176–82. – appears to be directly relevant. Please consider discussing it.

3. YgfJ is quite clearly a nucleotidyltransferase of dinucleotide sugar transferase Rossmanoid fold (verify by PSIBLAST or HHPred). This can be worked into the pathway reconstruction.

#### Authors' response

The highly compressed format of the Discovery Note departs somewhat from the style of longer papers. We have clarified the discussion of the most likely alternative hypothesis, namely that TIGR03309 members are accessory proteins for molybdenum hydroxylases that detoxify selenate in a process that further requires activation by SelD, rather than accessory proteins for molybdenum hydroxylases that contain selenium. This alternative hypothesis does not seem strong because the molybdopterin-containing enzymes that accompany TIGR03309 are varied and do not lead to the identification of protein families exclusively in SelD-positive genomes; only our putative accessory protein families, TIGR03309 in particular, do that. On the other hand, our proposed role for TIGR03309 in SDMH maturation suggests a labile selenium-dependent enzyme in *E. coli*, something not yet observed, so alternative hypotheses must be considered.

The note on animals with selenophosphate synthetase but no selenocysteine is consistent with several scenarios, the most likely being a role in the selenouridine modification to tRNA, which does occur in animals. A selenium-dependent molybdenum hydroxylase system is possible, but TIGR03309 and other putative SDMH markers hit no animal proteins.

The molybdenum-containing cofactors in selenium-dependent and other molybdenum hydroxylases are molybopterin derivatives, complex in their structure and variable between enzymes. In the SDMH nicotinic acid hydroxylase from *E. barkeri*, for example, Gladyshev and Lecchi identified molybdopterin cytosine dinucleotide. A nucleotidyltransferase role is therefore fully consistent with a role for YgfJ and other members of TIGR03310 as SDMH accessory proteins.

### Reviewer 2: Kira S. Makarova, National Center for Biotechnology Information

In this manuscript several considerations are described in support of the hypothesis that selenophosphate synthase SelD is not only the necessary component of selenouridine and selenocysteine systems but is also involved in formation of (or activation the selenium for) molybdenum hydroxylases, some of which are shown experimentally to be selenium-dependent.

The observation that two (out of ~500 analyzed) organisms with completely sequenced genomes contain the selD-like gene but neither genes for selenouridine and selenocysteine systems prompted the authors to investigate the neighborhood of these "orphan" selD in these two genomes in order to find a system where the SelD protein may function in these organisms. In the close vicinity of selD gene the homologs of the selenium dependent molybdenum hydroxylases (SDMH) can be found in both genomes suggesting a possible link between SelD and SDMH in these two genomes. This led the authors to hypothesis that these enzymes are likely selenium-dependent and SelD might be involved in the selenium activation for these enzymes.

One way to further support this hypothesis is to consider the inferred operon organization of the SelD-SDMH genome neighborhood. If selD could be predicted to co-transcribe with SDMH genes it would lend additional weight to the author's arguments. However these genes are co-directed and separated by a short (<100 bp) intergenic spacers only in *E. faecalis *while the neighborhood pattern in *H. marismortui *is incompatible with co-transcription and/or co-regulation. This lack of conservation of the selD-SDMH neighborhood weakens the evidence of their functional connection. It will be interesting to check all other genomes containing both selD and SDMH genes if there are any other cases where these genes might be inferred as co-transcribed or co-regulated.

Co-occurrence is another way to predict a functional link between genes, but it is particularly hard to use this evidence for functional linking of protein families, especially for those with large number of paralogs. The authors admit that the Phylogenetic Profiling methods rely on "good" protein families, but do not provide any considerations if the families or subfamilies investigated here are "good" or "bad". In this respect it is better not to go into details describing the advantages or drawbacks of the particular method but rather make a safe statement that PPP results are used essentially for enriching the set by the genes with possible functional link for further analysis. The parameters of the PPP search which can be used for reproducing the reported results are however of interest.

It should be explicitly stated why the authors do not show all the top 25 results of Partial Phylogenetic Profiling but only some selected subset. Apparently they do not believe that the omitted genes are relevant to selenium metabolism. Ideally, both the original data and the author's rationale for including or discarding a gene should be available to readers.

The strongest evidence that is presented in the paper is the notion that TIGR03309 is found only in the genomes containing SelD. Since the genomes that encode for TIGR03309 family proteins belong to a variety of bacterial clades there is a little doubt that the connection between SelD and TIGR03309 family is real and strong. But is this connection direct or indirect? If most of these organisms share an ecological niche or lifestyle it is possible that they would share a number of functionally unrelated genes due to shared physiologic requirements or simply physical proximity.

The authors explain the observed connection between SelD and TIGR03309 family by hypothesizing that TIGR03309 proteins recruit selenophosphate for use in SDMH system. Are there other reasonable alternative hypotheses? How do the authors discriminate between them?

I believe however that despite the relatively weak support, the hypothesis described here is interesting, entirely reasonable and is an excellent starting point for further investigation by both experimental and computational means.

#### Authors' response

Dr. Makarova is correct to point out that the circumstantial evidence supporting TIGR03309 as a marker for selenium dependent molybdenum hydroxylases, though substantial, is not yet sufficient to eliminate doubt. We formed our hypotheses based on the number of genomes we could investigate with PPP, a tool that exploits pre-computed all-vs.-all BLAST comparison results. We waited, however, until more whole genome shotgun sequences became available so we could test TIGR03309 against SelD to a much greater depth in non-redundant sequences. These added genomes include *Clostridium phytofermentans *ISDg, the third instance of an orphan SelD genome, taxonomically quite different from the other two. In genomes with either selenocysteine or selenouridine systems, *selD *sits far from the TIGR03309 family gene, but in *C. phytofermentans*, the three occur in the gene order *selD *– TIGR03527 – TIGR03309, and once again near xanthine dehydrogenase homologs. This is the third different arrangement for selD and TIGR03309 in three orphan SelD genomes (one gene between, three genes between, and adjacent but converging), but they are always close. We are not troubled by the lack of conserved operon structure because there is no reason to expect similar expression levels of SelD and TIGR03309 to be required. We have somewhat expanded the discussion of *C. phytofermentans*.

The taxonomies of TIGR03309-containing species are quite diverse, but so are their environmental niches. Several represent human intestinal bacteria, while others include a Dead Sea halophilic archaeon, a carbon monoxide-metabolizing thermophile, a ubiquitous water-borne mesophile, a metal-reducing delta proteobacterium, etc. We think it unlikely the link to SelD represents an indirect link to a common environmental factor.

We agree with Dr. Makarova's assertion (except for top hits with stand-out statistical significance) that the PPP algorithm does not guarantee a meaningful link between a protein family and a biological process, but rather enriches for proteins likely to be relevant. PPP results may reflect both false-positives and false-negatives for proteins that are not simultaneously required for and restricted to the occurrence of a system in a genome. Table 1 therefore cleans the signal slightly by imposing two requirements. Proteins are shown if they are identifiable as parts of conserved gene regions shared with E. coli (top 17 rows) and E. faecalis (bottom 3 rows), and their PPP rank is shown if in the top 25. We have tried to make the legend for table 1 clearer.
